# Forty-one degrees celsius enhances proliferation of chicken muscle satellite cells via mechanistic target of rapamycin activation and mitochondrial metabolism^☆^

**DOI:** 10.1016/j.psj.2025.105875

**Published:** 2025-09-20

**Authors:** Dongjin Yu, Leecheon Kim, Jongryun Kim, Junseok Ban, Kwanseob Shim, Darae Kang

**Affiliations:** a Department of Animal Resources and Biotechnology, College of Agriculture Life Science, Jeonbuk National University, Jeonju 54896, Republic of Korea; b Department of Animal Biotechnology, Jeonbuk National University, Jeonju 54896, Republic of Korea; c Department of Agricultural Convergence Technology, Jeonbuk National University, Jeonju 54896, Republic of Korea; d Institute of Agricultural Science and Technology, Jeonju 54896, Republic of Korea

**Keywords:** Chicken muscle satellite cell, Cultured meat, Proliferation, Temperature, Mitochondrial metabolism

## Abstract

Chicken muscle satellite cells (**CMSCs**) possess a self-renewal capacity and myogenic differentiation potential, making them valuable cellular resources for cultured meat production. Enhancing the proliferation rate of CMSCs is essential to improve production efficiency, and cellular proliferation is highly sensitive to changes in the culture temperature. Generally, the standard culture temperature for most cells is 37 °C. however, this does not reflect the physiological body temperature of chickens. In this study, we cultured CMSCs at 37, 39, 41, and 43 °C to determine the optimal temperature for proliferation and investigate the metabolic responses of cells under these conditions. Cell counting and CCK8 assays revealed that CMSCs cultured at 41 °C exhibited a significantly higher proliferation rate than those cultured at other temperatures. Furthermore, compared to the 37 °C control group, cells cultured at 41 °C showed enhanced mitochondrial function and increased adenosine triphosphate (**ATP**) production, accompanied by the upregulation of genes associated with the mechanistic target of rapamycin (**mTOR**) signaling pathway. Although reactive oxygen species (**ROS**) generation was elevated at 41 °C, no significant change in the expression of the antioxidant enzyme superoxide dismutase 1(**SOD1**) was observed, and the expression of Catalase decreased. Additionally, no significant differences were observed in the expression of apoptotic pathway-related factors. These findings suggest that 41 °C is the optimal temperature for promoting the proliferation and mitochondrial metabolism of CMSCs, providing insights into the optimization of culture conditions for cultured meat production.

## Introduction

Satellite cells are skeletal muscle stem cells found in muscle tissues that play key roles in the regeneration and maintenance of damaged muscle fibers (Seale et al., 2001). In the quiescent state, satellite cells remain undifferentiated and are temporarily arrested during the cell cycle. Upon muscle injury, they are activated, proliferate, and differentiate into multinucleated myofibers (Kuang et al., 2007). Satellite cells are critical cellular resources for cultured meat research because of their self-renewal and differentiation capabilities.

Cultured meat refers to meat produced using stem cell-based technologies and is regarded as a promising alternative to address the challenges of conventional livestock production and meet the growing global demand for protein (Choi et al., 2021; Post, 2012). One of the key factors in cultured meat production is the efficient proliferation of satellite cells. Various approaches have recently been undertaken to optimize cell growth and culture conditions (Ahmad et al., 2023; Kolkmann et al., 2022; Yun et al., 2023). Culture temperature has been shown to significantly influence cell proliferation and metabolic regulation (Harding et al., 2016). Mammalian cells are typically cultured at 37 °C, which reflects their physiological temperature (Rieder and Cole, 2002). However, chickens maintain a higher internal body temperature of 41 to 42 °C in adulthood, and even during embryonic development, metabolic activity raises the internal temperature by approximately 1–1.5 °C above the optimal incubation temperature of 37.5–37.8 °C (Troxell et al., 2015; YALCIN et al., 2022). Given these physiological differences, it is questionable whether the standard culture temperature of 37 °C fully reflects the physiological characteristics of chicken muscle satellite cells (**CMSCs**). Recent studies have shown that culturing CMSCs derived from the leg muscle of day 19 Brown line embryos at 41 °C significantly enhances their proliferation (Kim et al., 2023), and similar results were observed in CMSCs derived from the pectoral muscle of day 18 Ross embryos (Gregg et al., 2023). These findings suggest that a culture temperature of 41 °C may positively influence the proliferative capacity of CMSCs.

Changes in CMSC proliferation are closely associated with mitochondrial energy metabolism (Antico Arciuch et al., 2012). Mitochondria are the primary source of intracellular adenosine triphosphate (**ATP**) and play a central role in regulating energy metabolism via oxidative phosphorylation and glycolysis (Nunnari Suomalainen, 2012). Alterations in culture temperature may modulate mitochondrial biogenesis, thereby affecting cell proliferation (Liu Brooks, 2012). However, excessive metabolic activity increases the generation of reactive oxygen species (**ROS**), potentially leading to cellular damage. Therefore, elucidating the effects of culture temperature on CMSC proliferation and mitochondrial metabolism is essential for optimizing cultured meat production.

In this study, CMSCs were cultured at four different temperatures (37, 39, 41, and 43 °C), and mitochondrial function, ROS production, antioxidant gene expression, and apoptotic markers were compared based on the temperature that showed the highest proliferation rate. Using this approach, we aimed to identify the optimal temperature conditions that maximize CMSC proliferation and metabolic activity, thereby providing fundamental insights into the development of efficient cultured meat production strategies.

## Materials and methods

### Cell culture and temperature treatment

Chicken muscle satellite cells isolated from the leg muscles of 18-day-old chick embryos (Ross 308), as described in a previous study (Park et al., 2024), were provided by the Department of Animal Biotechnology, Jeonbuk National University. The cells were cultured in DMEM/F-12 medium (11320082, Gibco) supplemented with 15 % FBS (16000044, Gibco), 1 % penicillin/streptomycin (BS-0081R, Gibco), and 1 % l-glutamine (25030-081, Gibco), 10 ng bFGF (233-fb-500/CF, R&D systems). Cells were seeded onto culture plates and allowed to attach at 37 °C for 24 h. The cells were then subjected to temperature treatments at 37 °C (control), 39 °C, 41 °C, or 43 °C for 48 h.

### Cell viability assay

Cell viability was assessed using the CCK8 assay (CK04-11, Dojindo, Kumamoto, Japan) 24 and 48 h after temperature treatment. Cells were seeded at 1.32 × 10^4^ cells/well in 96-well plates and cultured at 37 °C for 24 h for cell attachment. The cells were then cultured at 37, 39, 41, and 43 °C for 24 and 48 h and treated with CCK8 solution according to the manufacturer's instructions. After 3 h of incubation at each temperature, the absorbance was measured at 450 nm using a Microplate Reader (Multiskan Go, N10588, Thermo Fisher Scientific, Waltham, MA, USA).

### Cell proliferation assay

For proliferation analysis, cells were seeded at a density of 8.6 × 10⁴ cells/well in 6-well plates. After a 24 h attachment period at 37 °C, the temperature treatments were applied for 24 and 48 h. Cells were stained with Acridine Orange/Propidium Iodide (F23001, Logos Biosystems) and counted using a LUNA-FL Dual Fluorescence Cell Counter (L20001, Logos Biosystems).

### Immunocytochemistry

The cells were fixed with 500 μL of 4 % paraformaldehyde (**PFA**) at 4 °C for 20 min and washed thrice with PBS containing 0.3 % Triton X-100. Blocking was performed with 3 % BSA in DPBS for 1 h at room temperature. Cells were incubated overnight at 4 °C with the following primary antibodies: PAX7 (ab528428, DSHB, IA, USA; 1:50) and MyoD1 (18943-1-AP, Proteintech, IL, USA; 1:200). After washing, Alexa Fluor 488 (A-11001, Thermo Fisher Scientific, 1:1000) and Alexa Fluor 568 (A-11011, Thermo Fisher Scientific, 1:1000) secondary antibodies were added and incubated for 2 h at room temperature. Nuclei were stained with 4′,6-diamidino-2-phenylindole (**DAPI**, D9542, Sigma-Aldrich, St. Louis, MO, USA, 1:1000) for 5 min. Images were captured using a Leica DFC 9000 microscope (Leica, Wetzlar, Germany).

### Cell cycle and ROS measurement

Cell cycle and ROS analyses were performed after culturing at 37 °C for 24 h and exposing the cells to 37 °C and 41 °C for 48 h. For cell cycle analysis, cells were fixed in cold 70 % ethanol for 5 min, washed twice with PBS containing 1 % BSA, and stained with 200 μg/mL RNase A (70856, Sigma-Aldrich, MO, USA) and 20 μg/mL propidium iodide (**PI**, 421301, BioLegend, CA, USA). For intracellular ROS analysis, cells were incubated with 10 μM CM-H₂DCFDA (C6827, Invitrogen) in HBSS without phenol red (14025092, Gibco) for 30 min at the respective temperature. After staining with 1 μg/mL PI for 5 min, the samples were analyzed using flow cytometry.

### Western blot analysis

Following the temperature treatment, the cells were washed three times with cold PBS, and proteins were extracted using RIPA buffer. Protein concentrations were measured using a DC Protein Assay Kit (5000111, Bio-Rad). Equal amounts of protein were separated by 12 % sodium dodecyl sulfate-polyacrylamide gel electrophoresis and transferred to PVDF membranes. After blocking with 3 % skim milk for 1.5 h, the membranes were incubated overnight at 4 °C with the following primary antibodies: Pax7 (1:1000), MyoD (1:2000), Caspase 3 (bs-0081R, Bioss, 1:2000), and GAPDH (MA5-15738, Invitrogen, 1:5000). After washing, the membranes were incubated with goat anti-mouse secondary antibodies (31430, Thermo Fisher Scientific, Waltham, MA, USA) for Pax7 and GAPDH, and goat anti-rabbit secondary antibodies (31460, Thermo Fisher Scientific, Waltham, MA, USA) for MyoD and Caspase 3. Protein bands were detected using an ECL kit (34580; Thermo Fisher Scientific, Waltham, MA, USA) and visualized using an iBright CL100 imaging system (Thermo Fisher Scientific, Waltham, MA, USA). Band intensities were normalized to those of GAPDH and expressed as relative values.

### Quantitative real-time PCR

Total RNA was extracted using an AccuPrep Universal RNA Extraction Kit (K-3140, BIONEER, Daejeon, Republic of Korea) according to the manufacturer's instructions. RNA concentration and purity were assessed using a microvolume spectrophotometer (K12C-KR-001 W; KLAB, Daejeon, Republic of Korea). cDNA was synthesized using the AccuPower CycleScript RT PreMix (K-2047-B, BIONEER, Daejeon, Republic of Korea). qRT-PCR was performed using AccuPower 2X GreenStar qPCR Master Mix (K-6253, BIONEER, Daejeon, Republic of Korea) on a CFX Duet Real-Time PCR System (12016265, Bio-Rad Laboratories, Hercules, CA, USA). cDNA was denatured at 95 °C for 5 min, followed by 40 cycles of denaturation at 95 °C for 5 s and annealing at 60 °C for 5 s. Relative gene expression was normalized to GAPDH and calculated using the 2^−ΔΔCt^ method (Livak Schmittgen, 2001). The primers used for the housekeeping and target genes are listed in Table 1.

**Table 1:** Primer sequences used in quantitative real-time PCR.

Gene name	Accession number	F/R	Sequence (5′→3′)	Product size (bp)
GAPDH	NM_204305.2	F	GGACGCTGGGATGATGTTCT	292
		R	GGGTGGTGCTAAGCGTGTTA	
Pax7	NM_205065.1	F	AGGTACCAAGAGACGGGCTC	411
		R	CTCGGCAGTGAAAGTGGTCC	
MyoD	NM_204214.3	F	CGCAGGAGAAACAGCTACGA	138
		R	ACATGTGGAGTTGTCTGTGGA	
CCNE1	NM_001031358.2	F	TATCCACCAAAGTTGCACCA	247
		R	CCAGCACACAGAGATCCAAG	
CCND1	NM_205381.2	F	GCACAGCAGCACAACGTATC	84
		R	ATCTCGCACATCAGTGGGTG	
CCNA2	NM_205244.3	F	TCAGCGATATCCACACGTACC	99
		R	CCCGCATGTTGTTGGTGATG	
E2F1	NM_205219.2	F	GGAATGGGTGCTGTGGGAGAT	253
		R	AGCCAGGGAGGAGGAAACAAAC	
MKi67	XM_040674400.2	F	GCAACAACAAGGAGGCTTCG	204
		R	TTCAGGTGCCATCCCGTAAC	
SOD1	NM_205064.2	F	TTGTCTGATGGAGATCATGGCTTC	98
		R	TGCTTGCCTTCAGGATTAAAGTGAG	
Catalase	NM_001031215.2	F	ACCAAGTACTGCAAGGCGAAAGT	91
		R	ACCCAGATTCTCCAGCAACAGTG	
Caspase3	NM_204725.2	F	AGGATGCTGAAGGAACACGC	71
		R	GCCACTCTGCGATTTACACG	
Bcl2	NM_205339.3	F	ATCGTCGCCTTCTTCGAGTT	150
		R	ATCCCATCCTCCGTTGTCCT	
PI3K	NM_001004410.2	F	ACAATGCCATCTTACTCCAGG	125
		R	ACATAGGTTGCACAGAGGATTC	
AKT	NM_001396387.1	F	CATTCCCGCCATTATGAATGAAGTA	130
		R	CTTGTAGCCAATGAATGTGCCATC	
mTOR	XM_040689168.2	F	AAGGTTTCTTCCGGTCCATATC	98
		R	ATCAGGCCAGTGACCATAATC	
S6K1	NM_001030721.2	F	CATGATTTCCAAACGACCAGA	134
		R	AGTAAACCAAACAAGCCCTCC	
4E-BP1	XM_424384.8	F	ACCAGGATTATTTATGACCG	174
		R	TTCACCTACATTCGCTTTCT	

Abbreviations: GAPDH, glyceraldehyde-3-phosphate dehydrogenase; Pax7, paired box 7; MyoD, myogenic differentiation; B-cell lymphoma-2, Bcl2; PI3K, phosphatidylinositol 3-kinase; AKT, protein kinase B; S6K1, ribosomal protein S6 kinase; 4E-BP1, Eukaryotic translation initiation factor 4E binding protein 1;.

### Measurement of oxygen consumption rate (OCR)

OCR was analyzed using a Seahorse XF96 Pro extracellular flux analyzer (S7850A, Agilent, Santa Clara, CA, USA) with the Cell Mito Stress Test Kit (103015-100, Agilent) according to the manufacturer’s instructions. CMSCs were seeded at a density of 8 × 10³ cells/well and incubated at 37 °C for 24 h, followed by an additional 48-h incubation at either 37 °C or 41 °C. The plate was then transferred to an XF96 analyzer, and sequential injections of oligomycin (1 μM/well), FCCP (0.5 μM/well), and rotenone/antimycin A (0.5 μM/well) were administered. OCR was measured at the respective treatment temperatures (37 °C or 41 °C). Data were analyzed using the Seahorse XF-96 Wave Pro software and normalized to the total protein content using a DC Protein Assay Kit.

### Statistical analysis

All statistical analyses were performed using SAS (version 9.4). Data are presented as mean ± standard error of the mean (SEM). One-way analysis of variance (ANOVA), followed by Duncan’s multiple range test, was used to determine statistical differences among the temperature treatment groups. An independent *t*-test was performed to compare the statistical differences between the 37 °C control and 41 °C treatment groups. Statistical significance was set at *p* < 0.05 for all analyses.

## Results

### Effects of temperature treatment on proliferation and viability

**Fig. 1:**
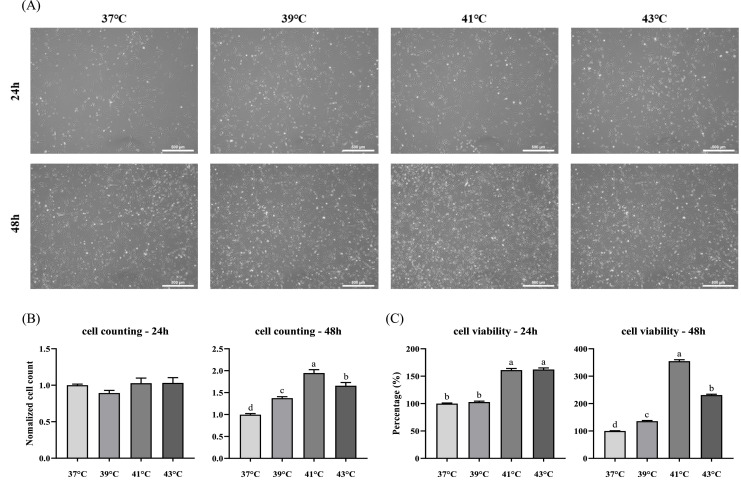
Effect of temperature treatment on the proliferation and viability of chicken muscle satellite cells (CMSCs). (A) Morphology, (B) cell counting, and (C) cell viability according to temperature and time elapsed after cell attachment (24 h). Values are expressed as mean ± SE. ^a-d^ indicate significant differences (*p* < 0.0001). Scale bar indicates 500 μm.

To identify the optimal culture temperature for CMSC proliferation, cells were incubated at various temperatures, and proliferation was evaluated via morphological observation, cell counting, and CCK8 assays. No morphological differences were observed between the groups (Fig. 1A). Cell counting showed no significant difference at 24 h, but significantly higher cell numbers were observed at 41 °C after 48 h of treatment than in the other groups (*p* < 0.0001) (Fig. 1B). CCK8 analysis revealed that cell viability was significantly higher at 41 °C and 43 °C after 24 h, with the highest viability observed at 41 °C after 48 h (*p* < 0.0001) (Fig. 1C).

### Effects of temperature treatment on myogenic transcription factor expression

**Fig. 2:**
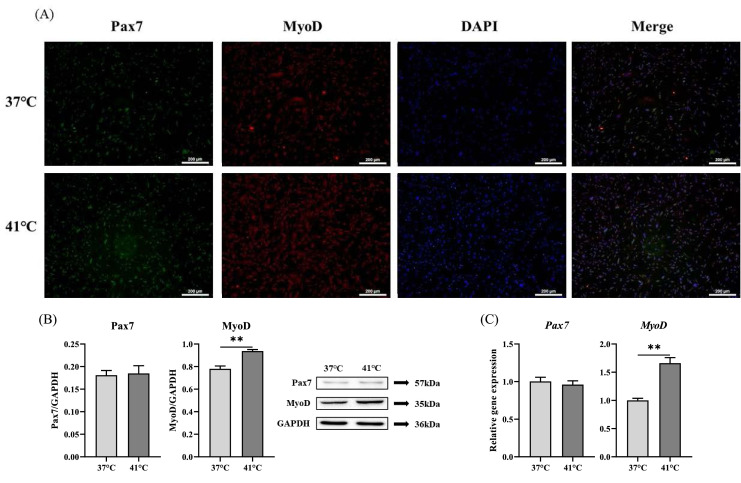
Effect of temperature treatment on the expression of myogenic transcription factors in chicken muscle satellite cells (CMSCs). (A) Immunofluorescence staining images of myogenic transcription factors in CMSC. Cell nuclei were stained with DAPI (blue), Pax7 (green), and MyoD (red). (B) Protein expression levels of Pax7 and MyoD. (C) Relative mRNA expression of *Pax7* and *MyoD*. Values are expressed as mean ± SE. ** Indicates significant difference between the treatment and control groups (*p* < 0.01). Scale bar indicates 200 μm.

To investigate the effects of temperature on myogenic transcriptional regulators, we analyzed the expression of Pax7 and MyoD, which are associated with satellite cell maintenance and myogenesis. Protein expression of Pax7 did not differ significantly between the groups. however, MyoD expression was significantly upregulated in the 41 °C group (*p* < 0.01) (Fig. 2A, B). Similarly, *Pax7* expression was not significantly different between the treatment and control groups, whereas *MyoD* expression significantly increased in the 41 °C group (*p* < 0.01) (Fig. 2C).

### Effects of temperature treatment on cell cycle

**Fig. 3:**
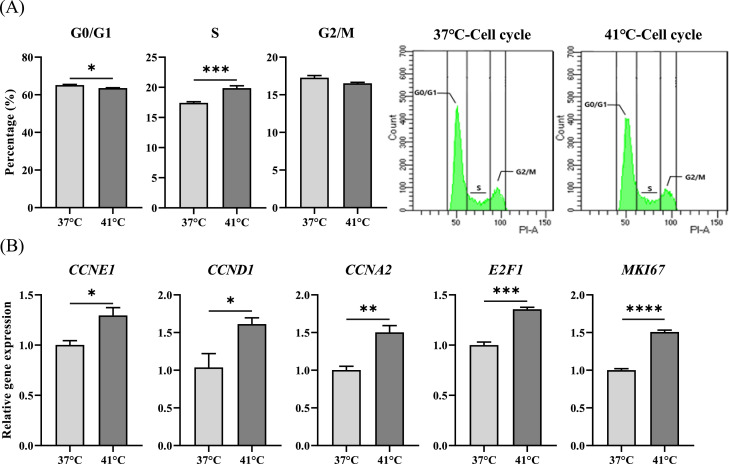
Effect of temperature treatment on the cell cycle of chicken muscle satellite cells (CMSCs). (A) Changes in the cell cycle according to the culture temperature in CMSCs. (B) Relative mRNA expression of *CCNE1, CCND1, CCNA2, E2F1,* and *MKI67*. Values are expressed as mean ± SE. *, **, ***, **** Indicate significant differences between the treatment and control groups (*p* < 0.05, *p* < 0.01, *p* < 0.001, *p* < 0.0001).

Cell cycle analysis revealed a significant decrease in the G0/G1 phase (*p* < 0.05) and an increase in the S phase (*p* < 0.001) in the 41 °C group, with no significant change observed in the G2/M phase (Fig. 3A). Additionally, gene expression analysis of cell cycle regulators showed significant upregulation of *CCNE1, CCND1, CCNA2, E2F1,* and *MKI67* at 41 °C (*p* < 0.05) (Fig. 3B).

### Effects of temperature treatment on intracellular ROS, antioxidants, and apoptosis

**Fig. 4:**
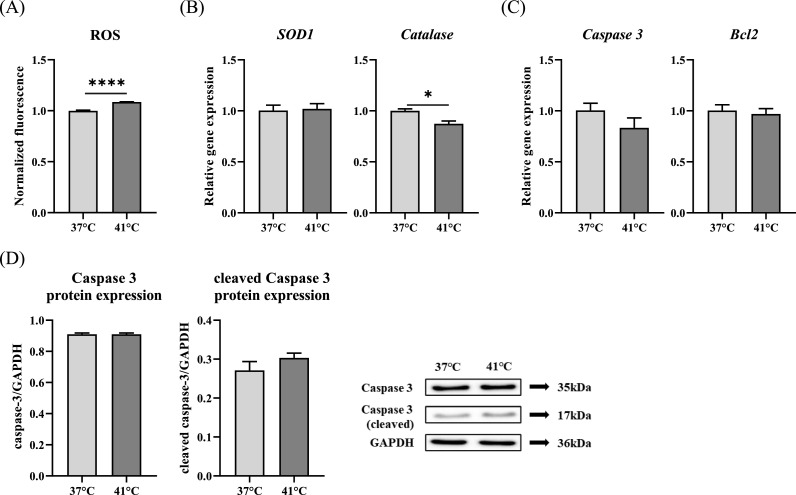
Effect of temperature treatment on intracellular reactive oxygen species (ROS) levels and apoptosis in chicken muscle satellite cells (CMSCs). (A) Intracellular ROS production following temperature treatment. (B) Relative mRNA expression levels of *SOD1* and *Catalase*. (C) Relative mRNA expression of *Caspase 3* and *Bcl2*. (D) Caspase 3 and cleaved Caspase 3 protein expression. Values are expressed as mean ± SE. *, **** Indicate significant differences between the temperature treatment and control groups (*p* < 0.05, *p* < 0.0001).

Intracellular ROS levels were significantly elevated in the 41 °C group (*p* < 0.0001) (Fig. 4A). To evaluate antioxidant responses, the gene expression levels of *SOD1* and *Catalase* were analyzed. *SOD1* expression remained unchanged, whereas *Catalase* expression was significantly reduced in the 41 °C group (*p* < 0.05) (Fig. 4B). To determine whether increased ROS levels induced apoptosis, we assessed the expression of apoptosis-related genes and proteins. There were no significant differences in the expression of *Caspase 3* and *Bcl2* genes (Fig. 4C), and there were no significant differences in the expression of Caspase 3 protein and cleaved Caspase 3 protein between the treatment and control groups (Fig. 4D).

### Effects of temperature treatment on mitochondrial metabolism

**Fig. 5:**
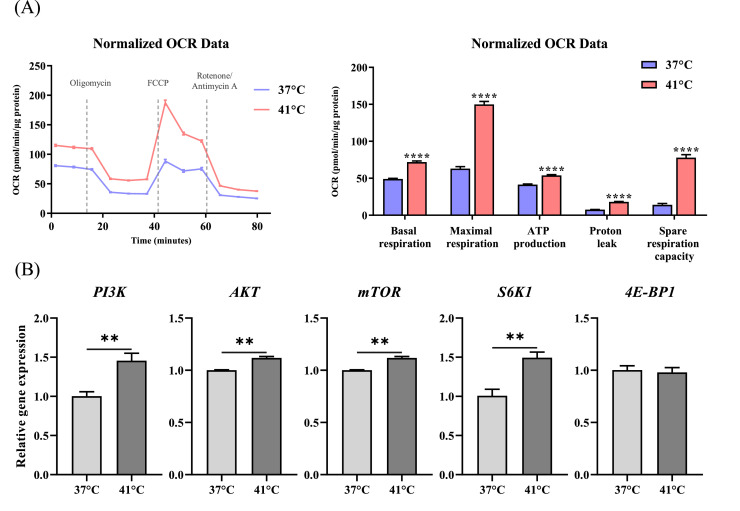
Effect of temperature treatment on mitochondrial metabolism. (A) Mitochondrial parameters, including oxygen consumption rate (OCR), in chicken muscle satellite cells (CMSCs) after 48 h of temperature treatment. (B) Relative mRNA expression levels of *PI3K, AKT, mTOR, S6K1*, and *4E-BP1*. Values are expressed as mean ± SE. **, **** Indicate significant differences between the temperature-treated and control groups (*p* < 0.01, *p* < 0.0001).

Mitochondrial function was assessed by measuring the OCR. Basal respiration, maximal respiration, ATP production, proton leak, and spare respiratory capacity were significantly increased in the 41 °C group (*p* < 0.0001) (Fig. 5A). Gene analysis of the mechanistic target of rapamycin (**mTOR**) pathway revealed that the expression of *PI3K, AKT, mTOR,* and *S6K1* significantly increased in the 41 °C group (*p* < 0.01). In contrast, the expression of *4E-BP1* did not differ significantly between the groups (Fig. 5B).

## Discussion

In cultured meat production, the efficiency of muscle stem cell proliferation is directly linked to productivity. Among the various culture conditions, temperature plays a pivotal role in modulating cell proliferation (Harding et al., 2016). Therefore, identifying the optimal temperature for muscle stem cell culture is a key strategy for improving the production efficiency. In this study, we aimed to determine the optimal culture temperature for promoting the proliferation of chicken muscle satellite cells and to investigate the influence of temperature on cell proliferation by analyzing intracellular ROS levels and mitochondrial metabolism.

To identify the optimal temperature for proliferation, CMSCs were cultured at 37, 39, 41, and 43 °C. Among these, 41 °C significantly enhanced cell proliferation and viability, consistent with previous studies (Gregg et al., 2023; Kim et al., 2023). Based on these results, we selected 41 °C as the treatment condition for subsequent analyses and compared it with the standard 37 °C control.

CMSCs are finely regulated during proliferation and differentiation by the action of myogenic transcription factors. Pax7 is a muscle cell-specific marker and transcription factor that determines the fate of self-renewal and muscle differentiation (Oustanina et al., 2004). MyoD is a transcription factor belonging to the Myogenic Regulatory Factor (**MRF**) family and is a marker of active satellite cells and myogenic commitment (Gerhart et al., 2007; Koishi et al., 1995). The expression patterns of these transcription factors are useful indicators for evaluating the cell state. In this study, Pax7 expression was not significantly different between the groups, whereas MyoD expression was significantly elevated in the 41 °C group. MyoD interacts with the Cyclin D1/CDK4 complex to generate high mitogenic signals that promote Rb phosphorylation, leading to the activation of E2F1 and subsequent G1/S phase progression (Bertoli et al., 2013; Wei Paterson, 2001). In the 41 °C group, the expression of key genes regulating the G1/S transition and cell cycle progression increased, and the proportion of S phase cells also increased, as determined by cell cycle analysis. These results suggest that the 41 °C culture conditions may have created an environment that induced the activation and proliferation of CMSCs.

Mitochondria are central to energy production, generating ATP through oxidative phosphorylation and regulating various metabolic reactions (Nunnari Suomalainen, 2012). Adequate energy supply is essential for cell proliferation, and mitochondrial energy metabolism plays a central role in meeting this energy demand (Zhu Thompson, 2019). Basal and maximal respiration reflect the energy demand and metabolic activity of the cell, and spare respiration capacity is used as a marker of mitochondrial fitness in muscle cells that consume large amounts of energy (Marchetti et al., 2020). In the 41 °C group, basal respiration, maximal respiration, and spare respiration capacity were significantly increased. These results suggest that culturing at 41 °C enhanced mitochondrial OCR, thereby promoting metabolic activity and creating an environment capable of meeting the energy demands required for cell proliferation. mTOR is a key protein kinase that regulates various cellular processes, such as growth and proliferation, and is activated in response to the intracellular energy status (Dennis et al., 2001). The 41 °C group showed a significant increase in ATP production, which reflects the amount of oxygen consumed for ATP synthesis, along with the elevated expression of genes related to the mTOR pathway. This suggests that increased ATP generation at 41 °C activates the mTOR pathway, leading to the upregulation of downstream targets such as S6K1 and Cyclin D1, thereby promoting cell proliferation. This enhanced metabolic activity is closely associated with ROS generation, and intracellular ROS levels were significantly increased at 41 °C.

ROS are reactive oxygen species generated when electrons leak from the electron transport chain during mitochondrial oxidative phosphorylation and react with oxygen (Venditti et al., 2013). Excessive ROS levels can lead to macromolecular damage and trigger apoptosis (Redza-Dutordoir Averill-Bates, 2016). To protect against oxidative damage, cells employ mechanisms such as proton leak to reduce ROS production and antioxidant systems to neutralize ROS (Brookes, 2005; Goel et al., 2021). In our study, ROS levels and proton leak were significantly elevated at 41 °C, but *SOD1* expression remained unchanged and *Catalase* expression decreased. This suggests that although ROS production increased, the antioxidant system did not respond robustly. This suggests that the activation of proton leak in response to increased ROS may have suppressed further ROS production to a certain level, thereby preventing the induction of the antioxidant system (Nanayakkara et al., 2019), or that elevated ROS-induced cellular damage may have inhibited the activation of the antioxidant system (Huang et al., 2015; Ibtisham et al., 2018).

To determine whether ROS accumulation at 41 °C caused cellular damage, we analyzed the expression of apoptosis-related genes and proteins. Activated Caspase 3 is a major executor of apoptosis that induces cell death by cleaving specific substrates (Porter Jänicke, 1999). Bcl2 regulates mitochondrial membrane integrity and has anti-apoptotic functions (Brunelle Letai, 2009). The analysis showed no significant changes in the expression of apoptosis-related genes and proteins in the 41 °C group. This suggests that the increased ROS levels in the 41 °C group did not cause cellular damage and were not high enough to activate the antioxidant response. As a limitation, the activation of the mTOR pathway in this study was evaluated only at the transcriptional level. Future studies should include protein-level validation, such as Western blotting with antibodies against chicken mTOR and phosphorylated mTOR, to provide more direct evidence of pathway activation.

## Conclusion

In this study, we investigated the optimal culture temperature for the proliferation of CMSCs and comprehensively analyzed how this temperature affected cellular metabolism, ROS levels, antioxidant systems, and apoptosis. Cell proliferation was significantly increased at 41 °C, which appeared to be due to enhanced mitochondrial metabolism and activation of the mTOR signaling pathway. Although ROS levels were elevated at 41 °C, the increase in proton leakage effectively regulated the accumulation of ROS. *SOD1* expression showed no significant difference compared to the control group, *Catalase* expression was reduced, and no significant changes were observed in the expression of genes or proteins related to the apoptosis pathway. These findings suggest that 41 °C is a stable condition that promotes CMSC proliferation without causing cellular damage. This study provides valuable foundational data for optimizing the culture conditions of CMSCs for meat production.

## CRediT authorship contribution statement

**Dongjin Yu:** Writing – review & editing, Writing – original draft, Methodology, Investigation, Formal analysis, Data curation, Conceptualization. **Leecheon Kim:** Visualization, Methodology. **Jongryun Kim:** Visualization, Software. **Junseok Ban:** Resources. **Kwanseob Shim:** Project administration. **Darae Kang:** Writing – review & editing, Supervision, Project administration, Funding acquisition, Conceptualization.

## Disclosures

The authors declare that they have no known competing financial interests or personal relationships that could have appeared to influence the work reported in this paper.
